# CAR-T Cell Therapy for HIV Cure: Current Challenges, Advances and Future Directions

**DOI:** 10.3390/v17121615

**Published:** 2025-12-14

**Authors:** Monica-Daniela Padurariu-Covit, Costinela Georgescu, Mihaela Andreescu, Iulia Chiscop, Catalin Plesea-Condratovici, Manuela Arbune

**Affiliations:** 1 Department of Clinical Medicine, Medicine and Pharmacy Faculty, “Dunarea de Jos” University of Galati, 800008 Galati, Romania; monica.padurariu@ugal.ro (M.-D.P.-C.); manuela.arbune@ugal.ro (M.A.); 2 Department of Hematology, Emergency County Hospital Sf. Apostol Andrei, 800578 Galati, Romania; 3 Department of Pharmaceutical Sciences, Medicine and Pharmacy Faculty, “Dunarea de Jos” University of Galati, 800008 Galati, Romania; 4 Faculty of Medicine, Titu Maiorescu University, 031593 Bucharest, Romania; tevetmihaela@gmail.com; 5 Department of Hematology, Colentina Clinical Hospital, 050098 Bucharest, Romania; 6 Department of Clinical Surgery, “Dunarea de Jos” University of Galati, 800008 Galati, Romania; iulia.chiscop@ugal.ro; 7 Department of Morphological and Functional Sciences, Faculty of Medicine and Pharmacy, “Dunarea de Jos” University of Galati, 800008 Galati, Romania; catalin.plesea@ugal.ro; 8 Department of Infectious Diseases, Clinic I, Infectious Diseases Clinic Hospital Sf. Cuv. Parascheva, 800179 Galati, Romania

**Keywords:** HIV cure, CAR-T cells, gene editing

## Abstract

Antiretroviral therapy (ART) effectively suppresses HIV replication but fails to eradicate latent reservoirs, leading to viral rebound after interruption. Chimeric antigen receptor (CAR) T-cell therapy offers a potential strategy to achieve durable remission. A systematic PubMed search (July 2020–June 2025) identified 253 studies on CAR-T therapy in HIV; 74 met inclusion criteria and were qualitatively analyzed. Preclinical data showed that CAR-T cells can recognize and eliminate infected cells, reach viral reservoirs, and persist long term, particularly when derived from hematopoietic stem cells. Dual-target and combination approaches with checkpoint inhibitors or latency-reversing agents enhanced antiviral efficacy. Early clinical studies confirmed safety and modest reservoir reduction. CAR-T cell therapy represents a promising step toward a functional HIV cure. Further optimization of design, integration with gene-editing technologies, and standardized clinical evaluation are required to confirm durable efficacy and safety.

## 1. Introduction

The pandemic caused by the human immunodeficiency virus (HIV) was first identified in the early 1980s and has affected more than 75 million people worldwide [1]. Susceptibility to HIV infection is generally universal, with the exception of individuals carrying the homozygous CCR5Δ32/Δ32 mutation, estimated to occur in 1–3% of the population, predominantly among Caucasians. Mutations in this gene protect cells from viral entry, particularly CD4^+^ helper T lymphocytes (LTCD4^+^), whose infection requires activation of surface receptors through one or both cellular coreceptors, CCR5 and CXCR4 [2].

Combination antiretroviral therapy (ART) has advanced remarkably, achieving durable suppression of plasma viral replication, improving survival, and transforming HIV infection into a chronic, manageable disease. Nevertheless, the complete eradication of infection remains an unfulfilled goal due to the persistence of the virus within latent viral reservoirs. These reservoirs consist of CD4^+^ T lymphocytes into whose DNA the virus integrates and remains as a permanent cellular component [3].

ART requires lifelong administration and strict adherence; discontinuation inevitably results in viral rebound originating from the latent viral reservoir. Ongoing research is exploring alternative therapeutic strategies aimed at achieving sustained virologic suppression in the absence of ART [4]. Before the advent of combination antiretroviral therapy, numerous non-ARV strategies were explored in attempts to control HIV infection through modulation of the immune system or reduction in viral load. Early approaches included the administration of interleukin-2 (IL-2), designed to stimulate CD4^+^ T-cell proliferation, and interferon-α, used to enhance endogenous antiviral responses. Although these therapies produced transient increases in CD4^+^ T-cell counts, large clinical trials (ESPRIT and SILCAAT) failed to demonstrate significant clinical benefit, leading to their discontinuation as therapeutic options [5,6].

With the discovery of viral latency in long-lived CD4^+^ T cells [7], attention shifted toward strategies designed to eliminate or control these reservoirs. In this context, modern immunotherapies such as broadly neutralizing antibodies (bNAbs) and therapeutic vaccines have demonstrated the ability to transiently reduce viral replication and strengthen immune control [8,9]. One emerging strategy aims to induce deep silencing of latent HIV reservoirs using latency-inducing or latency-promoting agents. Suppression of HIV transcriptional machinery prevents proviral reactivation but often fails to maintain long-term suppression after therapy discontinuation. Reprogramming the chromatin environment during proviral suppression could sustain a deeply silenced state, thus preventing viral rebound following ART interruption. This process could be reinforced by enhancing host immune recovery [10,11,12].

HIV-specific chimeric antigen receptor T-cell (CAR-T) therapy represents a promising option for achieving a functional cure for HIV infection in people living with HIV (PLWH), through the reactivation and elimination of latently infected cells. For successful clinical translation, anti-HIV CAR-T cells must be capable of trafficking to lymphoid tissues and eradicating reactivated HIV-infected targets such as CD4^+^ lymphocytes and monocytes/macrophages [13]. The concept of chimeric antigen receptor (CAR) T cells was first introduced in the 1980s by Eshhar and Gross, with the aim of redirecting T-cell responses through genetic engineering of T-cell receptors (TCRs). The first clinical application of this novel technology was explored as a potential therapy for HIV infection, in which cytotoxic CD8^+^ T lymphocytes (CTLs) were genetically modified to express CD4—the natural ligand of HIV—as an extracellular domain, linked to a transmembrane region and an intracellular CD3ζ signaling domain [14].

Although early CD4-based CAR constructs demonstrated proof of concept, their therapeutic impact was limited by poor in vivo expansion, lack of costimulatory domains, and vulnerability of the engineered cells to HIV infection. These challenges prompted significant technological evolution, including the incorporation of second- and third-generation signaling domains, improvements in viral vector design, and gene-editing strategies to enhance resistance to HIV. These advances—largely driven by breakthroughs in the oncology field—directly shaped the modern generation of HIV-directed CAR-T strategies currently under investigation [15].

In modern medicine, CAR-T cell therapy represents a revolutionary advance in the treatment of hematologic malignancies, relying on the genetic modification of T lymphocytes to specifically target and destroy tumor cells. A key component of this technology is the use of viral vectors that enable precise delivery of genetic material into host cells. Among these, lentiviruses and retroviruses are particularly important due to their ability to stably integrate the desired genes into the genome of target cells [16].

Lentiviral and retroviral vectors enable stable gene integration and have been widely used in CAR-T manufacturing. Lentiviral vectors can transduce both dividing and non-dividing cells, while retroviral vectors require cell proliferation [17,18,19].

Eight documented cases of HIV cure achieved through allogeneic hematopoietic stem cell transplantation (allo-HSCT) have provided crucial insights into mechanisms relevant for CAR-T development. These include CCR5 disruption, graft-versus-reservoir effects, and profound immune reconstitution, demonstrating that durable remission can occur through immune-mediated clearance of infected cells [20,21,22,23,24,25,26,27,28,29,30]. Cases of functional remission following ART interruption, including post-treatment controllers and elite controllers, further highlight the potential of immune-based strategies to maintain virologic control in the absence of therapy [31,32,33]. These insights have directly informed current CAR-T engineering approaches aimed at enhancing reservoir clearance and achieving sustained antiretroviral-free remission [34].

Following treatment interruption, several cases of functional remission have also been reported, in which the virus remains undetectable in blood but persists in latent form under immune control. The VISCONTI cohort (ANRS PRIMO/CO6) demonstrated that early ART initiation can enable long-term virologic control without therapy, while additional clinical studies have described rare post-treatment controllers who maintain suppression after ART discontinuation [31]. An aggregated analysis of 14 clinical studies conducted by the AIDS Clinical Trials Group (ACTG) identified 67 individuals who maintained virologic suppression without ART, including 38 who initiated treatment during early infection and 25 during the chronic phase, illustrating the existence of a rare subgroup of post-treatment controllers [32]. Another rare subgroup, termed elite controllers, comprises individuals who spontaneously maintain virologic suppression without ART, due to robust immune responses and extremely low viral reservoirs [33].

A highly promising strategy involves the use of allogeneic CAR-T cells, derived from healthy donors, an emerging off-the-shelf strategy, but clinical translation requires strategies to prevent graft-versus-host and host-versus-graft reactions. Gene editing to remove endogenous TCRs and the use of alternative lymphocyte platforms such as NK cells are under active investigation to improve safety and efficacy [35,36,37].

In this review, we first outline the virological and immunological basis for HIV persistence and the rationale for redirecting the immune response through CAR-T cell therapy. We then synthesize preclinical in vitro and in vivo evidence on CAR-T construct design, antiviral efficacy, reservoir targeting, and gene-editing strategies, followed by a summary of the available clinical and translational data in people living with HIV. Finally, we discuss the main technological, ethical, and operational challenges that currently limit widespread clinical implementation and highlight future directions for integrating CAR-T approaches into comprehensive HIV cure strategies.

## 2. Materials and Methods

### 2.1. Search Strategy

We registered the study protocol in the PROSPERO database with number CRD42041178068, to ensure methodological transparency and adherence to established standards for systematic reviews [38].

A systematic search of the scientific literature was performed to identify reported cases of HIV-positive patients who achieved viral cure and met our inclusion criteria. The search terms were used both as keywords and in combination as MeSH terms to maximize the retrieval of relevant studies. We conducted a comprehensive search in the PubMed database for articles published within the last five years (July 2020–June 2025), using the following algorithm:

(HIV OR HIV-1 OR HIV-2 OR “HIV cure” OR “functional cure” OR “HIV remission” OR “HIV eradication” OR “HIV healing” OR “HIV sterilizing cure” OR “HIV viral suppression without ART” OR “HIV cure cases” OR “HIV remission without ART” OR “HIV cured patients” OR “HIV remission patient” OR “HIV eradicated” OR “HIV remission treatment” OR “HIV remission therapy” OR “long-term viral suppression” OR “sustained remission”) AND (CAR OR “CAR-T cells” OR “CAR T cells” OR “Chimeric Antigen Receptor T cells” OR “CAR-modified T cells” OR “CAR-engineered T cells” OR “CAR-NK cells” OR “CAR-Macrophages” OR “CAR-targeted therapy” OR “Adoptive T-cell therapy with CAR” OR “Gene-modified T-cell therapy”).

This search yielded 253 PubMed records published between July 2020 and June 2025.

To structure the research question, we used the PICO framework, defining the population of interest as HIV-positive individuals included in clinical or preclinical studies involving CAR-T cell therapy.

### 2.2. Eligibility Criteria and Data Extraction

#### 2.2.1. Inclusion Criteria

For this review, studies were selected according to predefined inclusion criteria designed to capture the most relevant and scientifically significant contributions to the field of CAR-T cell therapy in the context of HIV infection.

We included original research articles describing preclinical studies (both in vitro and in vivo), as well as clinical trials in phases I, II, or III. In addition, systematic reviews, meta-analyses, editorials, and perspective papers discussing CAR-T cell therapy for HIV were also considered when they provided meaningful insights into this field.

The target population consisted of HIV-infected individuals at any stage of disease—latent, active, or virally suppressed under ART. The search was further extended to include animal model studies relevant to HIV pathogenesis and in vitro experiments using HIV-infected or genetically modified cell lines that simulate infection.

The intervention of interest was CAR-T cell therapy, whether specifically targeting HIV or including universal/allogeneic CAR-T strategies or combination therapies with other modalities such as antiretroviral therapy (ART) or gene-editing technologies. Studies investigating related approaches such as CAR-NK, CAR-macrophage, or other CAR-based cell therapies were also included.

Particular attention was given to studies reporting outcomes related to the efficacy of CAR-T therapy, including reductions in viral load or elimination of the latent HIV reservoir. We also extracted data on safety, tolerability, mechanisms of action, and challenges such as CAR-T cell exhaustion or viral immune evasion.

Studies addressing the use of CAR-T therapy in malignant diseases were included only if they provided relevant translational insights applicable to HIV treatment—such as shared mechanisms of immune escape, treatment efficacy, or innovative therapeutic strategies.

To reflect the most recent advances in the field, only studies published within the last five years and written in English were included to ensure consistency and accuracy in data interpretation.

Data were extracted systematically, focusing on study design, population characteristics, CAR-T therapy type, main outcomes, and reported limitations, in order to provide a clear and comprehensive overview of the challenges and progress made toward the use of CAR-T cell therapy as a potential approach to HIV cure.

#### 2.2.2. Exclusion Criteria

Several exclusion criteria were applied to ensure the inclusion of only high-quality and relevant studies.

Studies lacking sufficient information regarding the efficacy or safety of CAR-T therapy in HIV were excluded, as were general or theoretical papers that did not focus directly on evaluating CAR-T cell therapy in the context of HIV infection.

However, some articles that did not directly address HIV infection but provided valuable insights into the fundamental mechanisms of CAR-T therapy were retained.

Studies with weak methodological design were excluded, as well as those published in languages other than English (unless official translations were available), to ensure uniformity and accurate data interpretation.

In terms of intervention type, we excluded studies that did not focus on CAR-T therapy but rather on other cellular immunotherapies that did not involve CAR modification.

Non-original publications—such as commentaries, conference abstracts, or general reviews—were also excluded to maintain a focus on primary research of direct relevance.

After applying these exclusion criteria, 74 studies met the inclusion requirements and were retained for full analysis. These criteria were designed to ensure that only high-quality and scientifically relevant studies were included in the final synthesis.

### 2.3. Quality Assessment

The quality of the included studies was assessed using standardized tools appropriate for each study design. For non-randomized studies, we used the Risk of Bias in Non-Randomized Studies of Interventions (ROBINS-I) tool, and for randomized controlled trials, we applied the Revised Cochrane Risk-of-Bias tool (RoB 2) [39,40].

Although ROBINS-I and RoB 2 are widely used and validated tools for assessing risk of bias in interventional studies, their application in the context of HIV-directed CAR-T research has certain limitations. The small number of available studies, heterogeneous methodologies, variability in CAR construct design, and differences in outcome reporting may reduce the sensitivity of these tools to detect specific biases relevant to cellular immunotherapies. Additionally, many CAR-T studies are early-phase or exploratory studies, which can challenge the applicability of conventional bias-assessment frameworks.

Two independent reviewers (M.And. and I.C.) assessed each study across seven domains, including bias due to confounding, participant selection, intervention classification, deviations from intended interventions, missing data, outcome measurement, and selective reporting.

To ensure the accuracy and reliability of the assessment, two additional reviewers (C.G. and I.C.) independently cross-checked the evaluations. Any discrepancies identified during this process were resolved through discussion and consensus with the first author.

## 3. Results

**Figure 1:**
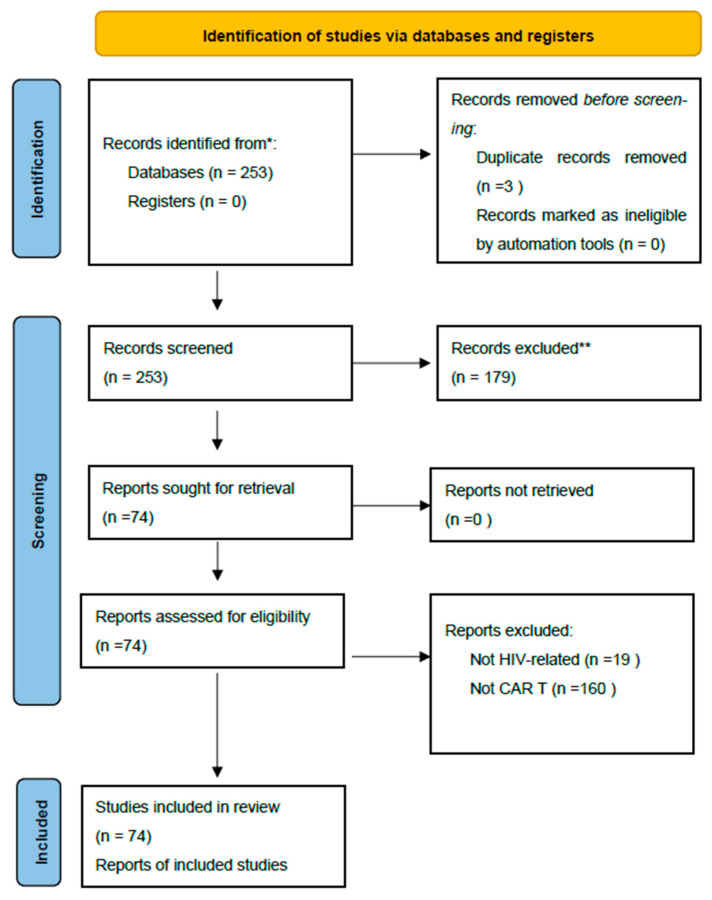
PRISMA Diagram [41]. * Consider, if feasible to do so, reporting the number of records identified from each database or register searched (rather than the total number across all databases/registers). ** If automation tools were used, indicate how many records were excluded by a human and how many were excluded by automation tools.

A total of 74 articles published between 1 July 2020 and 30 June 2025 were included in the final analysis. Of these, 25 were reviews, 37 were preclinical studies (in vitro and animal models), 12 were clinical studies (including case reports, small case series, and early-phase clinical trials), and 3 publications reported false-positive HIV test results following CAR-T therapy (Figure 1—PRISMA diagram).

The review articles primarily addressed the theoretical foundations and recent advances in CAR-T cell therapies, both in the context of HIV infection and hematologic malignancies, highlighting shared mechanisms and translational barriers to clinical application.

The preclinical studies (Table 1) represented the largest portion of the included literature, investigating the feasibility of cellular engineering, CAR-T cell persistence and expansion, and their efficacy in eliminating HIV-infected cells. Most studies employed lentiviral vectors for T-cell transduction, while several explored innovative constructs, including CAR-NK and CAR-macrophage platforms.

The clinical studies (*n* = 12) comprised case reports, small patient series, and one phase I clinical trial. These demonstrated the feasibility and safety of administering CAR-T cells to patients with HIV, with no severe adverse events reported. CAR-T cell persistence was generally transient, and virological control was limited, although in some cases a reduction in the viral reservoir was observed.

An important methodological consideration is represented by the three studies reporting false-positive HIV test results in patients treated with CAR-T cell therapy for hematologic malignancies, attributed to the use of lentiviral vectors. While these studies did not evaluate the efficacy of CAR-T therapy against HIV itself, they are crucial for the accurate interpretation of virological findings and for distinguishing true HIV infection from genetic therapy-induced artifacts.

The general characteristics of the included studies a and the main findings are presented in detail by category in Table 1, Table 2 and Table 3.

A total of 25 review articles on CAR-T cell therapy in the context of HIV infection were identified (Table 1).

A total of 37 preclinical studies were analyzed, providing insights into CAR-T design optimization, antiviral efficacy, and immune modulation strategies (Table 2).

Twelve clinical studies were identified: one CAR-T trial conducted in PLWH, seven CAR-T studies unrelated to HIV infection, and four case reports describing false-positive HIV nucleic acid test results following CAR-T therapy (Table 3).

## 4. Discussion

### 4.1. The Need for a Curative Approach to HIV and the Therapeutic Potential of CAR-T Cells

Despite the remarkable progress achieved with antiretroviral therapy (ART) in controlling HIV infection, current treatments fail to eradicate the virus, and treatment interruption invariably leads to viral rebound. This limitation is primarily attributed to the latent viral reservoir, composed mainly of CD4^+^ T lymphocytes harboring integrated provirus, residing in lymph nodes, the gut, the central nervous system, and other anatomically protected compartments [21,44].

**Figure 2:**
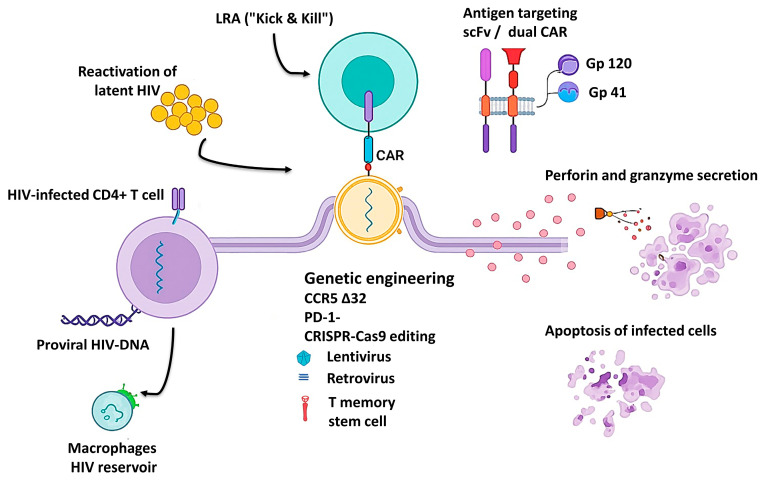
Schematic representation of CAR-T cell–mediated targeting of HIV reservoirs. The figure illustrates the main steps involved in CAR-T–based HIV eradication: latency reversal (“Kick & Kill”), recognition of HIV antigens by scFv- or dual-CAR constructs, genetic engineering approaches (CCR5Δ32 and PD-1 knockout), and cytolytic activity through perforin and granzyme secretion leading to apoptosis of infected cells. HIV reservoir components, including infected CD4^+^ T cells and macrophages, are also shown [14,44,46,56].

CAR-T cell therapy, which has revolutionized the treatment of several hematologic malignancies, offers an innovative strategy to redirect the immune system against HIV-infected cells. Specifically, this approach involves the genetic modification of T cells to express a chimeric antigen receptor (CAR) capable of recognizing viral antigens displayed on the surface of infected cells. As such, CAR-T therapy represents a promising candidate for achieving either functional or sterilizing HIV cure (Figure 2) [43,48].

Unlike ART, which maintains viral suppression without directly targeting infected cells, CAR-T–based therapies act independently of MHC expression, enabling the recognition and destruction of both latent and active HIV-infected cells [13,66]. Furthermore, newer generations of CAR-T cells incorporate HIV resistance mechanisms [48,80] and immune checkpoint blockade strategies [64], potentially enhancing persistence and curative potential.

A recent retrospective study from Romania analyzing 15 years of experience in treating HIV-associated lymphomas highlighted persistently high mortality rates and limited outcomes with conventional therapies, emphasizing the urgent need for innovative interventions such as CAR-T cell therapy [99].

### 4.2. Insights from Recent Literature on CAR-T Therapy in HIV Infection

#### 4.2.1. Mechanistic Advances

Recent literature reveals substantial refinement of CAR-T platforms, both in construct design and in the optimization of viral vectors used for gene transfer. Several comprehensive reviews [45,47,51] have described significant advances in lentiviral and retroviral vectors enabling stable CAR integration into T-cell genomes. A major focus has been placed on engineering HIV-resistant receptors through CCR5 gene knockout or by inserting CAR constructs directly into the CCR5 locus, thereby conferring dual protection—antiviral and self-protective (Figure 2).

Beyond these genetic innovations, recent studies have emphasized the inclusion of co-stimulatory domains (CD28, 4-1BB, ICOS) and mechanisms counteracting T-cell exhaustion, such as combining CAR-T therapy with immune checkpoint blockade (PD-1, CTLA-4). These improvements enhance persistence, proliferation, and cytotoxic function, enabling durable immune surveillance and specific targeting of HIV-infected cells (Figure 2) [66,67,87].

Another conceptual milestone is the development of dual-CAR and CAR-macrophage platforms capable of targeting multiple viral epitopes (e.g., gp120, gp41) or addressing tissue reservoirs inaccessible to conventional therapies (Figure 2). These hybrid designs represent a paradigm shift—from a purely cytolytic “kill-only” approach to a combined strategy of recognition, eradication, and long-term immune surveillance [14,44].

#### 4.2.2. Clinical and Translational Applications

Clinical reviews [37,52,53,54,55,56,58] highlight the transition of CAR-T from an oncologic innovation to a translational platform with significant potential in HIV, particularly in patients with HIV-associated lym1homas. These studies indicate that CAR-T cell administration in virally suppressed individuals under ART is feasible and safe, without major reactivation of viral replication.

Hattenhauer et al., 2023 [53] reported that HIV-positive patients can safely receive CAR-T therapy with outcomes comparable to those of HIV-negative individuals, provided that viral load and immune function are closely monitored. Similarly, Chen et al. [52] demonstrated that CAR-T therapy in HIV-associated lymphomas does not compromise virologic control and may even contribute to the elimination of infected cells within the tumor microenvironment.

An emerging clinical direction involves allogeneic “off-the-shelf” CAR-T therapies, using T cells from healthy donors genetically modified to prevent rejection and reduce manufacturing time [44,67]. This approach could transform CAR-T therapy from a highly individualized treatment into a standardized and scalable intervention, applicable even in chronic HIV infection.

#### 4.2.3. Challenges and Future Perspectives

Despite the exceptional promise of CAR-T therapy for eradicating HIV reservoirs, all major reviews converge on the existence of critical barriers hindering full clinical translation. The principal challenges include the anatomical sequestration of viral reservoirs, CAR-T cell exhaustion, anti-CAR immune responses, and the need for long-term persistence of the modified cells.

Ethical and social analyses [60] emphasize the complexity of patient acceptance of gene therapy among people living with HIV, particularly given ongoing stigma and fears surrounding genetic manipulation.

Recent perspectives [14,44,46] underscore the importance of combining CAR-T therapy with latency-reversing agents (LRA), checkpoint inhibitors, or CRISPR/Cas9-based editing, in a convergent effort to achieve functional or sterilizing cure. Thus, future directions clearly point toward integrated multimodal strategies addressing viral persistence, T-cell dysfunction, and long-term control of HIV replication.

### 4.3. Promising Preclinical Findings: Antiviral Efficacy and Reservoir Reduction

#### 4.3.1. Experimental Evidence of CAR-T Antiviral Activity

Preclinical studies consistently demonstrate that CAR-T therapy can efficiently recognize and eliminate HIV-infected cells, offering one of the most promising approaches for achieving functional cure. In humanized mouse models, CD4-based or dual-recognition (duoCAR) constructs have shown potent cytolytic activity against HIV-positive cells and significant reductions in plasma viremia (Figure 2) [13,66]. These findings were reinforced by sustained persistence of modified cells and retention of a central memory (TCM) phenotype—key for long-term viral control.

#### 4.3.2. Validation in Complex Animal Models

In non-human primate models, which closely mirror human immunology, convergent results have been observed. Barber-Axthelm et al. [69] and Carrillo et al. [70] demonstrated that CAR-T cells derived from hematopoietic stem cells migrate efficiently to deep lymphoid compartments, including B-follicular germinal centers—key sites of viral reservoirs. Their administration led to reduced proportions of HIV-infected CD4^+^ cells and partial decreases in tissue viral load, without inducing significant systemic toxicity. These findings suggest superior tissue trafficking and viral control compared to traditional CAR-T constructs.

#### 4.3.3. Therapeutic Synergies: CAR-T and Immune Checkpoint Blockade

Combining CAR-T therapy with checkpoint inhibitors (PD-1 or CTLA-4 blockade) has been shown to enhance antiviral activity and cell longevity. Eichholz et al. [65] and Pan et al. [67] demonstrated that this combination restores T-cell cytotoxicity, increases interferon-γ and granzyme B secretion, and reduces immune exhaustion, thereby optimizing the antiviral response.

#### 4.3.4. Optimization of Vectors and Genetic Engineering

Significant progress in lentiviral and retroviral vector design has improved both the safety and efficiency of CAR transduction. The series of studies by Urak [76,81,82] and Rothemejer [43] demonstrated the advantages of targeted CAR integration into the CCR5 locus, generating T cells resistant to HIV reinfection. This strategy provides dual protection by eliminating infected cells and preventing re-infection of engineered cells.

#### 4.3.5. Limitations, Causal Inference and Future Research

Despite encouraging preclinical data, clinical translation remains challenging. Limited CAR-T persistence, development of anti-CAR immune responses, and differences between animal models and human immunity constrain the interpretation of experimental outcomes.

In addition, early-phase studies often lack analytical treatment interruption, standardized quantification of replication-competent reservoirs, and longitudinal sampling strategies needed to evaluate durable effects on viral persistence [109,110].

A further limitation is the absence of methodological designs capable of supporting robust causal inference. Current findings remain largely associative and do not establish whether CAR-T therapy can causally reduce the latent reservoir or modify long-term viral dynamics [109]. Heterogeneity in virological endpoints, small sample sizes, and the lack of randomized or adequately controlled designs limit the ability to distinguish true CAR-T–mediated effects from confounding influences such as ongoing ART suppression or host-immune variability [111].

To overcome these limitations, upcoming phase I/II trials should adopt stepwise clinical designs that integrate CAR-T therapy with latency-reversing agents (LRAs) and PD-1 blockade, while simultaneously incorporating robust causal-inference methodologies—such as counterfactual modeling, longitudinal structural-equation approaches, and targeted maximum likelihood estimation [51,109]. These approaches will be essential to determine whether CAR-T therapy produces true causal reductions in the latent reservoir and contributes meaningfully to functional HIV cure efforts.

### 4.4. Clinical Perspectives on CAR-T Therapy in HIV

#### 4.4.1. Feasibility and Safety in People Living with HIV

Available clinical evidence supports the feasibility and safety of CAR-T therapy in people living with HIV (PLWH), particularly those with hematologic malignancies. Case series and clinical reports in lymphoma and multiple myeloma indicate that anti-CD19 and anti-BCMA CAR-T therapies can be administered safely without compromising viral control, provided that ART is maintained and carefully monitored [53,100,101]. The toxicity profile (CRS/ICANS) appears comparable to that observed in HIV-negative patients, though pre-existing immunosuppression may predispose to opportunistic infections—such as refractory cryptosporidiosis reported post-CAR-T therapy [105].

#### 4.4.2. Early Efficacy Signals in HIV-Focused Studies

The first dedicated clinical trial targeting HIV [97] demonstrated safety and tolerability of a multifunctional CAR-T platform (M10) and provided early indications of virologic control and CAR-T persistence in circulation. Although limited by small sample size and short follow-up, these findings support the translational rationale derived from preclinical studies and justify future phase II trials with standardized virological endpoints, including reservoir quantification.

#### 4.4.3. Clinical and Operational Considerations in PLWH

Clinical practice recommendations and narrative analyses emphasize the importance of maintaining ART throughout manufacturing and post-infusion, performing infectious screening and prophylaxis (HBV, CMV, Pneumocystis), managing comorbidities and drug–drug interactions (e.g., corticosteroids or tocilizumab for CRS), and selecting appropriate targets (CD19 vs. BCMA) with optimal timing for bridging therapy [36,53].

Allogeneic “off-the-shelf” strategies remain highly attractive due to reduced production time and product standardization but require additional mechanisms to prevent allo-reactivity and rejection prophylaxis [67].

#### 4.4.4. Virological Monitoring: The Pitfall of Post–CAR-T False Positives

Several reports have documented false-positive HIV-1 RNA/DNA detections following CAR-T therapy using lentiviral vectors due to cross-reactivity of certain nucleic acid amplification tests (NAATs) with vector-derived sequences [107,108,109]. These findings carry major clinical implications, underscoring the need for corroborative testing across multiple platforms (plasma RNA, cellular proviral DNA) and documentation of the specific vector sequence used in manufacturing. Accurate interpretation requires contextual analysis—absence of viremia on orthogonal assays and lack of clinical signs of viral rebound. Such considerations should be integrated into standard monitoring protocols for PLWH receiving CAR-T therapy to prevent misdiagnosis and unnecessary interventions.

#### 4.4.5. Gaps, Limitations, and Research Priorities

Although early clinical outcomes of CAR-T therapy in HIV infection are promising, the current evidence base remains fragmented and heterogeneous. Most studies involve small cohorts, variable designs, and non-standardized virological endpoints. The absence of analytical treatment interruptions (ATI) prevents assessment of sustained remission after ART withdrawal—a key criterion for defining functional cure [53].

To advance beyond the exploratory stage, standardized phase I/II clinical trials are required, employing robust virological markers such as ultrasensitive HIV RNA, integrated proviral DNA, and ex vivo inducibility assays. Integrated strategies combining CAR-T therapy with latency reversal (LRAs) and checkpoint blockade may offer synergistic benefits by simultaneously reducing the latent reservoir and restoring T-cell functionality [64,87].

Next-generation platforms, including allogeneic “off-the-shelf” CAR-T cells and CCR5-targeted integration constructs, promise enhanced persistence and improved safety profiles. Parallel development of specific monitoring guidelines is necessary to address HIV NAAT false-positivity and infection risk management in immunocompromised settings [15].

Overall, the transition from preclinical promise to clinical validation requires harmonized evaluation criteria and close multidisciplinary collaboration. Strengthening these directions will be pivotal for advancing CAR-T therapy from an experimental concept to a realistic therapeutic pathway toward functional HIV cure [70].

In addition to these barriers, current clinical trials also face methodological limitations. Virological endpoints remain heterogeneous across studies, with assays varying in sensitivity, specificity, and their ability to quantify the replication-competent reservoir. The absence of standardized outcome measures complicates cross-trial comparisons and limits the ability to determine true reductions in the latent reservoir. Furthermore, long-term follow-up data are largely unavailable, preventing definitive conclusions about the durability of CAR-T–mediated remission once ART is interrupted. These limitations underscore the need for harmonized virological criteria and extended follow-up in future CAR-T trials for HIV [110].

### 4.5. Technological Challenges: Persistence, Viral Evasion, and Safety

Although preclinical results and early clinical observations confirm the potential of CAR-T therapy in controlling HIV infection, its full translation into clinical practice remains limited by a series of complex technological and biological obstacles. These challenges can be grouped into three major dimensions: persistence of the modified cells, viral evasion, and therapeutic safety [69,72].

#### 4.5.1. Persistence and Expansion of CAR-T Cells

Insufficient persistence of CAR-T cells is one of the main obstacles to achieving durable viral control. In the absence of robust expansion and long-term survival, the therapeutic effect remains transient.

Studies conducted in SIV/HIV models [64,70,89] have shown that, although CAR-T cells can be detected in circulation for several weeks after infusion, their numbers decline progressively in the absence of constant antigenic stimulation.

An emerging direction consists of deriving CAR-T cells from hematopoietic stem/progenitor cells (HSPC), which provide a regenerative compartment with the potential for prolonged persistence [69].

In parallel, CRISPR/Cas9 gene editing has enabled integration of the CAR cassette into the CCR5 locus, conferring resistance of T cells to HIV infection and reducing the risk of virus-mediated depletion [80,81].

At the same time, immune checkpoint blockade (PD-1, CTLA-4) has proven a valuable adjunct strategy, prolonging CAR-T activity and proliferation by mitigating cellular exhaustion [64,67].

#### 4.5.2. Viral Evasion and HIV Adaptability

Immune escape remains one of HIV’s most sophisticated evolutionary strategies, enabling evasion of CAR recognition through modifications of viral epitopes.

Studies by Jiang [66] and Meng [78] demonstrated that mutations in the gp120 region can markedly reduce the affinity of CD4-based CARs, limiting lytic efficiency.

To counter this phenomenon, bispecific (dual-CAR) constructs have been developed that can recognize multiple viral epitopes simultaneously—or even both tumor antigens and HIV antigens [74,76].

More recently, synNotch platforms permit sequential activation of CAR-T cells only in the presence of specific combinations of antigens, reducing the risk that infected cells “escape” detection and limiting off-target effects [73,95].

In addition, combining CAR-T therapy with latency-reversing agents (LRAs) has proven promising for exposing latent cells to immune attack [39,78].

#### 4.5.3. Safety and Immunogenicity

Clinical experience in HIV is still limited; the extensive body of evidence in onco-hematology provides benchmarks for safety assessment.

No severe cases of cytokine release syndrome (CRS) or neurotoxicity have been reported in HIV-focused studies [72,97]. However, these risks remain possible, particularly with highly potent antiviral activity.

An emerging issue is the development of anti-CAR antibodies that can neutralize modified cells and limit therapeutic efficacy [63].

Moreover, the use of HIV-derived lentiviral vectors generates the risk of false-positive HIV NAAT results, a problem recently reported in clinical case studies [59,106]. These phenomena underscore the need for differentiated testing protocols capable of distinguishing non-infectious vector sequences from active viral genomes.

From an ethical and logistical perspective, Dubé [60] and Hattenhauer [53] draw attention to barriers in access to advanced therapies, high costs, and the need for specialized biosafety infrastructure.

Overall, although preclinical and early clinical studies support the feasibility and acceptable safety profile of CAR-T therapy in HIV infection, concerns related to long-term persistence, immune-mediated clearance of modified cells, and potential off-target or immunogenic effects continue to limit broader clinical translation [96,100].

### 4.6. Ethical Considerations in CAR-T Therapy

HIV-related stigma and discrimination continue to represent major barriers to accessing services for people living with HIV (PLHIV) worldwide. It is estimated that one in eight individuals living with HIV is denied healthcare services due to stigma and discrimination, while research indicates that more than 50% of people report holding discriminatory attitudes toward those living with HIV. The UNAIDS strategy identifies the elimination of stigma and the achievement of “zero discrimination” as essential goals in the effort to end the AIDS epidemic by 2030 [112].

Studies involving both patients and healthcare providers have shown that HIV-related stigma within oncology care settings decreases the likelihood that people living with HIV (PLWH) will receive any form of cancer treatment (chemotherapy, radiotherapy, or surgery), resulting in lower survival rates compared to their HIV-negative counterparts [113].

Oncologic therapies have inspired HIV cure strategies through the use of experimental cellular and gene-based approaches aimed at generating HIV-resistant cells, enhancing host immunity, or neutralizing the virus. CAR-T cell therapy holds curative potential by improving the adaptive immune response against HIV; however, it requires careful monitoring due to the risk of cytokine release syndrome and neurotoxicity. Moreover, some CAR-T clinical trial protocols involve structured interruptions of antiretroviral therapy (ART), representing one of the four major domains of normative ethics in HIV cure research—alongside the acceptable balance between benefit and risk, the ethical integrity of informed consent, and the consideration of infection risks to sexual partners [53,60].

The decision to administer CAR-T cell therapy to patients with HIV can be challenging given the large number of cellular therapy programs—including those targeting patients with severe disease—alongside the high costs and difficulties associated with producing these cellular products. Therefore, the prioritization of patients for CAR-T treatment is a complex process that must rely on ethical principles and procedurally fair criteria, taking into account the limited availability of treatment slots, as well as the patient’s estimated medical benefit, risk profile, and overall prognosis [114].

Transparency in the allocation of CAR-T cell therapy ensures the legitimacy of these innovative interventions and helps strengthen trust in both the research and clinical application of CAR-T therapy among people living with HIV.

## 5. Conclusions

Despite major advances in antiretroviral therapy (ART), the persistence of HIV within latent reservoirs continues to prevent complete viral eradication. Chimeric antigen receptor T-cell (CAR-T) therapy, originally developed for hematologic malignancies, has emerged as a promising strategy to redirect the immune system against HIV-infected cells.

A systematic analysis of the 76 included studies—encompassing preclinical, clinical, and translational data—highlights the consistent ability of CAR-T cells to recognize and eliminate HIV-infected CD4^+^ lymphocytes, achieve partial reduction of the viral reservoir, and demonstrate a favorable safety profile in early clinical investigations. Genetic engineering innovations, such as CAR integration into the CCR5 locus, bi-epitopic constructs, and stem cell-derived CAR platforms, have improved cellular persistence and antiviral potency. Moreover, combination strategies—such as pairing CAR-T therapy with immune checkpoint inhibitors or latency-reversing agents (LRAs)—may further enhance viral reservoir clearance.

Nevertheless, significant challenges remain, including the limited durability of CAR-T cells, the risk of immune rejection, and logistical barriers to clinical implementation. Overall, current evidence supports CAR-T therapy as a biologically active and safe intervention with curative potential, warranting validation in phase I/II clinical trials employing standardized virological criteria and analytical treatment interruption protocols to assess functional cure.

In the next decade, integrated CAR-T and gene editing strategies may redefine HIV management, transitioning from lifelong suppression to functional cure.

## Figures and Tables

**Table 1:** Summary of review articles on CAR-T cell therapy and HIV infection.

Category	Specific Category	No. of Articles	Type of Review	Main Focus/Observations
General CAR-T and HIV reviews	Overview	6 [4,14,20,42,43,44]	Narrative reviews	Broad perspectives on CAR-T applicability in HIV; discuss therapeutic challenges, feasibility, and future directions.
Mechanistic/Genetic engineering reviews	Mechanisms	8 [24,45,46,47,48,49,50,51]	Mechanistic/molecular reviews	Focus on CAR design, vector optimization, CRISPR-based editing, resistance mechanisms, and long-term persistence.
Clinical and translational reviews	Clinical	10 [33,37,52,53,54,55,56,57,58,59]	Clinical/translational narrative reviews	Discuss clinical application, safety, management of HIV + lymphomas, translational implications, and early clinical trials.
Ethical and social reviews	Ethical	1 [60]	Qualitative/ethical review	Examines patient perception, ethical implications, and social perspectives regarding cellular therapies in HIV.

**Table 2:** Mechanisms of action in CAR-T cell therapy for HIV—comprehensive (*n* = 37).

Category	Mechanism	No of Articles	Country	Study Type	Key Findings/Observations
Immunomodulation	Cytokine support (IL-15/IL-7)	1 [61]	USA	In vivo—macaques	IL-15 preconditioning enhanced CAR-T expansion and persistence.
	PD-1/checkpoint blockade	1 [62]	USA	In vivo—macaques	Revealed PD-1–associated immune exhaustion and latency maintenance in ART-treated macaques, supporting PD-1 blockade
	Immunogenicity/anti-CAR responses	1 [63]	USA	In vivo—macaques	Limited in vivo efficacy due to rapid CAR-T clearance.
	PD-1/checkpoint blockade	1 [64]	Multi-country	In vivo—macaques	Checkpoint blockade improved persistence and reduced T-cell exhaustion.
		3 [65,66,67]	China	In vitro/preclinical	Reducing PD-1 expression or blocking the PD-1 pathway significantly enhances CAR-T cell function against HIV by improving their expansion, strengthening antiviral activity, and increasing cytotoxicity and cytokine production.
	Immunogenicity/anti-CAR responses	1 [68]	USA	In vivo—macaques	Documented anti-CAR immune responses limiting persistence.
Stemness and persistence	HSPC-derived/long-lived CAR-T	2 [69,70]	USA	In vivo—macaques	Stem-cell–derived CAR-T maintained long-term persistence and tissue homing.
		2 [71,72]	USA [71], China [72]	In vivo—humanized mice (BLT)	Demonstrated that HSPC-derived CAR-T cells persist long-term, enhance viral control, and reduce the HIV reservoir; rapamycin improved CAR-T expansion and antiviral efficacy.
Targeting diversity	Bispecific/dual CAR constructs + Novel epitope targeting	5 [13,73,74,75,76]	USA	In vivo—humanized mice (BLT) [13,73]	Dual-target CAR-T recognized multiple HIV epitopes and limited viral escape.
				In vitro/preclinical [74,75,76]	New scFv binding domains improved antigen binding and signaling. Dual CARs targeting gp120/gp41 broadened antigen recognition.
Genetic protection	CCR5 editing/entry resistance	1 [77]	USA	In vivo—macaques	HSPC gene therapy with CCR5 editing conferred HIV entry resistance and included a safety kill switch for controlled elimination of transduced cells.
	Antiviral payloads	1 [78]	China	In vivo—humanized mice	Synthetic Notch–CAR-T cells secreted bNAbs upon HIV recognition, achieving durable viral control without ART.
	Vector safety/off-target	1 [79]	Canada	Mixed (in vitro + in vivo)	Assessed vector safety and off-target editing in preclinical assays.
	CCR5 editing/entry resistance	3 [80,81,82]	Netherlands [80]; USA [81,82]	In vitro/preclinical	CCR5 editing and vector engineering improved safety and resistance.
Reservoir targeting	Latency reversal + CAR-T (shock & kill)	1 [83]	USA	In vitro/preclinical	CAR-T efficiently targeted reactivated HIV-infected cells in latency models.
	Latency/reservoir interaction	1 [84]	Belgium	In vitro/preclinical	Validated CAR-T cytotoxicity after LRA-induced proviral reactivation.
	Latency reversal + CAR-T (shock & kill)	1 [85]	UK	In vitro/preclinical	Cleared reactivated reservoirs in primary CD4^+^ T-cell assays.
	Novel reservoir targeting	1 [86]	China	In vivo—humanized mice (BLT)	Combined novel targeting with latency modulation in BLT mice.
	Latency reversal + CAR-T (shock & kill)	1 [87]	China	Mixed (in vitro + in vivo)	LRAs combined with CAR-T enabled clearance of reactivated cells.
	Reservoir biology/latency	1 [88]	Italy	In vitro/preclinical	Explored CAR-T interactions with HIV latency dynamics.
Trafficking & homing	Tissue infiltration/lymphoid homing	2 [89,90]	Belgium [89]; Japan [90]	In vivo—macaque	Early viral control associated with improved CAR-T trafficking.
		1 [91]	USA	In vivo—humanized mice (BLT)	Evaluated cytotoxicity and persistence with tissue distribution metrics.
		1 [92]	USA	In vivo—macaque	Observed CAR-T infiltration in gut-associated lymphoid tissue (GALT).
Methods & manufacturing	Safety/cytokine profile	1 [93]	USA	In vitro/preclinical	Characterized cytokine release and safety parameters.
	Signaling strength/monitoring	1 [94]	USA	In vitro/preclinical	Refined monitoring and control of CAR signaling strength.
	Imaging/tracking methods	1 [95]	China	In vitro/preclinical	Developed imaging to monitor CAR-T distribution and dynamics.
Uncategorized		1 [96]	China	In vitro/preclinical	PD-1 knockdown improved CAR-T function against HIV targets.

**Table 3:** Clinical studies on CAR-T cell therapy in HIV and non-HIV populations.

Characteristics	No of Articles	Country	Study Type	Number of Patients	HIV Relevance	Key Findings/Observations
CAR T in HIV patients	1 [15]	China	Phase I, multicenter, single-arm trial	18 HIV-positive	Direct (anti-HIV M10 CAR-T)	First clinical trial of CAR-T in PLWH; demonstrated safety and viral suppression.
Non-HIV clinical studies	1 [97]	Romania	Retrospective, single-center	47 HIV-positive	Indirect (HIV-associated lymphomas, no CAR-T)	Contextual epidemiology of HIV-associated lymphomas; highlights need for innovative therapies.
	1 [98]	China	Clinical study, single center	11	Indirect (HIV-negative)	Evaluated efficacy and safety of CD19 CAR-T in relapsed/refractory B-cell lymphoma.
	1 [99]	China	Case report	1	Indirect (HIV-negative)	Long-term remission (5 years) after BCMA CAR-T therapy for multiple myeloma.
	1 [100]	China	Case report	1	Indirect (HIV-negative)	Reported complete remission of pancreatic cancer following CLDN18.2 CAR-T therapy.
	1 [101]	China	Case report	1	Indirect (HIV-negative)	Achieved sustained remission in DLBCL after CAR-T combined with bridging radiotherapy.
	1 [102]	Germany	Case report	1	Indirect (HIV-negative)	CAR-T feasible and effective in patient with GvHD post allogeneic SCT.
	1 [103]	USA	Case report	1	Indirect (HIV-negative)	Reported opportunistic cryptosporidiosis after CAR-T; highlights infection risks.
	1 [104]	Spain	Clinical methodological study	5 (B-ALL patients + 1 donor)	Indirect (HIV-negative)	Compared two flow-cytometry protocols for CAR-T cell monitoring; ligand-based assay showed higher sensitivity and good correlation with qPCR; useful for standardizing CAR-T follow-up.
Special aspects	4 [105,106,107,108]	USA	Case report	6	False-positive HIV tests after CAR-T therapy	Reports cases of false-positive HIV detection by PCR following CAR-T cell therapy, with important implications for clinical monitoring.

## Data Availability

All data supporting the reported results are included in the article.

## Abbreviations

The following abbreviations are used in this manuscript:

ACT	Antiretroviral combination therapy
allo-HSCT	Allogeneic hematopoietic stem cell transplantation
ATI	Analytical treatment interruption
bNAbs	Broadly neutralizing antibodies
BCMA	B-cell maturation antigen
BLT mice	Humanized bone marrow–liver–thymus mice
CAR	Chimeric antigen receptor
CAR-T	Chimeric antigen receptor T cells
CAR-NK	Chimeric antigen receptor natural killer cells
CCR5	C-C chemokine receptor type 5
CNS	Central nervous system
CRISPR/Cas9	Clustered regularly interspaced short palindromic repeats/Cas9 gene-editing system
CRS	Cytokine release syndrome
CTL	Cytotoxic T lymphocytes
CTLA-4	Cytotoxic T-lymphocyte antigen-4
CXCR4	C-X-C chemokine receptor type 4
DARPin	Designed ankyrin repeat proteins
duoCAR	Dual or bispecific chimeric antigen receptor
Env	HIV envelope glycoprotein
GALT	Gut-associated lymphoid tissue
GC	Germinal centers
GvHD	Graft-versus-host disease
HIV	Human immunodeficiency virus
HSPC	Hematopoietic stem/progenitor cells
HSCT	Hematopoietic stem cell transplantation
ICANS	Immune effector cell-associated neurotoxicity syndrome
ICOS	Inducible T-cell co-stimulator
IL-2/IL-7/IL-15	Interleukin-2/-7/-15
LRA	Latency-reversing agent
LV	Lentiviral vector
MHC	Major histocompatibility complex
MLV	Murine leukemia virus
NAAT	Nucleic acid amplification test
NHP	Non-human primates
NK	Natural killer cells
PD-1	Programmed cell death protein 1
PD-L1	Programmed death-ligand 1
PRISMA	Preferred Reporting Items for Systematic Reviews and Meta-Analyses
PROSPERO	International Prospective Register of Systematic Reviews
qPCR	Quantitative polymerase chain reaction
scFv	Single-chain variable fragment
SHIV	Simian–human immunodeficiency virus
SIV	Simian immunodeficiency virus
synNotch	Synthetic Notch receptor
TCM	Central memory T cells
TCR	T-cell receptor
TLR7	Toll-like receptor 7
4-1BB (CD137)	Costimulatory signaling domain 4-1BB
